# Duvelisib is a novel NFAT inhibitor that mitigates adalimumab-induced immunogenicity

**DOI:** 10.3389/fphar.2024.1397995

**Published:** 2025-01-09

**Authors:** Aboli Bhingarkar, Yuyin Wang, Keito Hoshitsuki, Katherine Marie Eichinger, Sanjay Rathod, Yin Zhu, He Lyu, Andrew T. McNutt, Larry W. Moreland, Lee McDermott, David R. Koes, Christian A. Fernandez

**Affiliations:** ^1^ Center for Pharmacogenetics and Department of Pharmaceutical Sciences, University of Pittsburgh School of Pharmacy, Pittsburgh, PA, United States; ^2^ Duo Oncology, Pittsburgh, PA, United States; ^3^ Department of Computational and Systems Biology, School of Medicine, University of Pittsburgh, Pittsburgh, PA, United States; ^4^ Division of Rheumatology, School of Medicine, University of Colorado, Aurora, CO, United States; ^5^ Department of Pharmaceutical Sciences, University of Pittsburgh School of Pharmacy, Pittsburgh, PA, United States

**Keywords:** rheumatoid arthritis, antidrug antibodies (ADA), NFAT (nuclear factor of activated T cells), methotrexate, adalimumab, duvelisib

## Abstract

**Introduction:**

TNFα inhibitor (TNFi) immunogenicity in rheumatoid arthritis (RA) is a major obstacle to its therapeutic effectiveness. Although methotrexate (MTX) can mitigate TNFi immunogenicity, its adverse effects necessitate alternative strategies. Targeting nuclear factor of activated T cells (NFAT) transcription factors may protect against biologic immunogenicity. Therefore, developing a potent NFAT inhibitor to suppress this immunogenicity may offer an alternative to MTX.

**Methods:**

We performed a structure-based virtual screen of the NFATC2 crystal structure to identify potential small molecules that could interact with NFATC2. For validation, we investigated the effect of the identified compound on NFAT transcriptional activity, nuclear localization, and binding to the NFAT consensus sequence. *In vivo* studies assessed the ability of the compound to protect against TNFi immunogenicity, while *ex vivo* studies evaluated its effect on CD4^+^ T cell proliferation and B cell antibody secretion.

**Results:**

We identified duvelisib (DV) as a novel NFATC2 and NFATC1 inhibitor that attenuates NFAT transcriptional activity without inhibiting calcineurin or NFAT nuclear localization. Our results suggest that DV inhibits NFAT independently of PI3K by interfering with nuclear NFAT binding to the NFAT consensus promoter sequence. DV significantly protected mice from adalimumab immunogenicity and attenuated *ex vivo* CD4^+^ T cell proliferation and B cell antibody secretion.

**Discussion:**

DV is a promising NFAT inhibitor that can protect against TNFi immunogenicity without inhibiting calcineurin phosphatase activity. Our results suggest that the future development of DV analogs may be of interest as agents to attenuate unwanted immune responses.

## Introduction

Immunogenicity is a characteristic of biological drugs that refers to their propensity to induce an immune response, leading to the production of anti-drug antibodies by the host and reducing the therapeutic efficacy of the agent. TNFα inhibitors (TNFi) are the most widely prescribed biologics in RA therapy (Meyer et al., 2021). Currently, there are five TNFα inhibitors approved for use in RA: infliximab, adalimumab, golimumab, etanercept, and certolizumab pegol (Findeisen et al., 2021). TNFi therapy is typically used following the failure of first-line therapy with methotrexate (MTX), which remains the standard of care for initial RA treatment. Although TNFi therapy has greatly improved RA outcomes, a significant number of patients experience treatment failure due to biologic immunogenicity (Souto et al., 2016). The process of anti-drug antibody formation is initiated by the uptake and processing of the biologic by antigen-presenting cells (APCs) and the presentation of the antigenic peptides on the major histocompatibility complex (MHC) class II molecules present on their cell surface. When the T cell receptor recognizes an antigen, helper T cells become activated and undergo rapid proliferation. Activated T cells release cytokines, such as CD40L, IFN-γ, IL-4, IL-5, IL-6, IL-10, IL-21 which activate B cells and promote release of antigen-specific antibodies and differentiation into plasma cells (Sauerborn et al., 2010; Doherty et al., 2018).

The concomitant administration of low-dose methotrexate (MTX) has been shown to suppress anti-drug antibody formation against TNFα inhibitors (Maini et al., 1998; Weinblatt et al., 2003). Previous studies have shown that MTX therapy induces the release of extracellular adenosine, which exerts an anti-inflammatory effect mediated by the activation of adenosine receptors (Cronstein et al., 1993; Montesinos et al., 2000; Montesinos et al., 2003; Friedman and Cronstein, 2019; Cronstein and Aune, 2020). However, while MTX has been shown to effectively reduce immunogenicity to TNFi biologics, the numerous adverse effects associated with the use of this cytotoxic agent are a major concern often resulting in MTX discontinuation (Alarcón et al., 1995; Kinder et al., 2005). Common adverse effects of MTX include gastrointestinal discomfort and stomatitis (Tsukada et al., 2013), hepatotoxicity (Sotoudehmanesh et al., 2010), and hematological disorders (Mameli et al., 2017). Given these limitations, alternative immunosuppressive agents, including azathioprine and calcineurin inhibitors like cyclosporine (CsA) and tacrolimus, can be considered potential options for mitigating biologic immunogenicity (Meneghini et al., 2021). Azathioprine suppresses T cell activation by inhibiting purine synthesis, while calcineurin inhibitors prevent T cell activation by inhibiting NFAT signaling, thereby reducing cytokine production and the activation of both T cells and B cells. However, these agents have significant toxicities that limit their use. For example, CsA is associated with neurotoxicity, nephrotoxicity, and cardiotoxicity (Farouk and Rein, 2020), while azathioprine has a toxicity profile similar to MTX, including hepatotoxicity, hematologic effects, and gastrointestinal toxicities (Dubinsky, 2004). Taken together, there is an urgent need to identify novel strategies that are well-tolerated by RA patients and as effective as MTX to optimize RA therapy and attenuate drug-induced immunogenicity.

In our previous research, we demonstrated that pharmacological inhibition of NFATC2 could protect against hypersensitivity reactions induced by another biologic, asparaginase (Rathod et al., 2020). Although current clinical and preclinical agents for inhibiting NFAT activation, such as cyclosporin A (CsA) and tacrolimus (FK506) (Flanagan et al., 1991), as well as the peptide inhibitor 11R-VIVIT (Aramburu et al., 1999), have shown effectiveness, their clinical use for preventing immunogenicity is limited due to toxicity or unfavorable PK properties, respectively. Therefore, there is a significant interest in identifying new and safe NFAT inhibitors that can potentially be used in the clinic.

The NFAT family of transcription factors comprises five distinct members that play a critical role in regulating gene expression in response to various cellular stimuli. Among these, NFATC1, NFATC2, NFATC3, and NFATC4 are activated by the calcium – calcineurin signaling pathway. Upon a rise in intracellular calcium levels, calcineurin phosphatase is activated, leading to the dephosphorylation of NFAT proteins. Consequently, these proteins translocate into the nucleus and bind to specific DNA regions, thereby regulating the expression of target genes (Rao et al., 1997; Lopez-Rodríguez et al., 1999). Furthermore, NFATC2 and NFATC1 are highly expressed NFAT transcription factors in T cells and play a crucial role in regulating cytokines that promote immune responses (Monticelli and Rao, 2002; Lee et al., 2018). In addition, NFATC2 and NFATC1 are essential for the expression of CD40L (Tsytsykova et al., 1996), CXCR5 (Vaeth et al., 2014), PD-1 (Oestreich et al., 2008) receptors, as well as cytokines such as IL-4 (Monticelli and Rao, 2002) and IL-21 (Kim et al., 2005), which are necessary for germinal center formation and B cell differentiation into plasma cells (Vaeth and Feske, 2018). Therefore, targeting NFAT could have a significant impact on T and B cell-mediated immune responses. Based on the need for novel alternative strategies to mitigate TNFi immunogenicity, we aimed to identify potential novel NFAT inhibitors. To achieve this, we performed a structure-based virtual screen to identify small molecules that could interact with NFAT and inhibit its function. Our approach focused on discovering compounds that effectively inhibit NFAT activity and protect against TNFi immunogenicity (Supplementary Figure S1). These tool molecules can be further developed in future studies to reduce immune responses and enhance the therapeutic efficacy of TNFα inhibitors in RA therapy.

## Materials and methods

### Computational analysis and molecular docking of NFATC2 for drug discovery

Molecular dynamics simulations of the DNA-bound NFATC2 crystal structure (PDBID: 1OWR) were performed to identify targetable transient pockets. Solvated periodic boxes of NFATC2, with and without its bound DNA, were simulated using AMBER20 with a 2 fs timestep. The simulation without DNA was parameterized with the ff15ipq force field for the protein and the TIP3P force field for the water and run for 60 ns (Jorgensen et al., 1983; Debiec et al., 2016). The simulation with DNA was parameterized with the ff14SB force field for the protein, the OL15 force field for DNA, and the TIP3P force field for water and run for 100 ns (Jorgensen et al., 1983; Maier et al., 2015; Galindo-Murillo et al., 2016). Additional simulation details, including RMSF, RMSD, radius of gyration, and surface area, are provided in Supplementary Figures S2, S3. For analysis, the simulations were downsampled to every 100ps and aligned to the N-terminal domain backbone of the starting structure after removing all non-protein atoms.

MDpocket was used to identify transient pockets that appeared during the simulation in the aligned and downsampled simulation (Schmidtke et al., 2011). After identifying pockets, fpocket was used to determine their druggability in each snapshot of the simulation (Le Guilloux et al., 2009; Schmidtke and Barril, 2010), and the top three druggable snapshots of the identified pockets were docked with probe molecules. Seven small-molecule probes (isopropanol, acetamide, acetate, isopropylamine, imidazole, isobutane, and benzene) were docked to these identified pockets using GNINA (McNutt et al., 2021). After docking, we manually curated the top-ranked probe poses to identify favorable interactions with pocket residues, and retained these probe poses for pharmacophore construction.

Pharmit was used to screen the MolPort database for potential ligands for each druggable snapshot of each pocket, using a radius of 1 Å for each identified pharmacophore feature, and accounting for the receptor shape (Sunseri and Koes, 2016). We manually selected pharmacophores using the identified probe interactions with the goal of selecting selective pharmacophore queries (approximately 200 hits). All hits were minimized and scored with GNINA to generate three affinity scores per molecule: Vina affinity and two convolutional neural network (CNN) computed affinities (CrossDock_Default 2018 and Dense) (Francoeur et al., 2020). Molecules were ranked based on their Vina affinity, two CNN-computed affinities, and overall ranking, and the top 31 commercially available compounds were selected for further evaluation.

### NFAT activation luciferase reporter assay

Jurkat-Lucia NFAT cells (InvivoGen) were used for the initial NFAT inhibitor screening experiments. Cells were cultured in complete medium consisting of IMDM supplemented with 1 mM L-glutamine, 25 mM HEPES, 10% heat-inactivated fetal bovine serum (FBS), and 100 μg/mL Normocin. To evaluate the impact of potential NFAT inhibitors on the activation of NFAT, 4 × 10⁵ cells suspended in 180 μL of medium were stimulated with PMA (50 ng/mL) and ionomycin (3 μg/mL) to induce NFAT activation, following the manufacturer’s instructions. These experiments were conducted in ninety-six-well microtiter plates (Corning, United States) with three biological replicates per condition to ensure data reliability and reproducibility. Prospective small molecule NFAT inhibitors were evaluated at a concentration of 10 µM (Supplementary Table S1), which was chosen to ensure a saturated dose before performing concentration-dependent studies to estimate potency. After 24 h, 20 µL of the supernatants were mixed with 50 µL/well of QUANTI-Luc luciferase substrate (InvivoGen) and luminescence was measured using a BioTec H1 Synergy reader (BioTek, Winooski, United States).

Cyclosporine A (CsA, MedChem Express, HY-B0579) was used as a positive control for NFAT inhibition, and a non-ionomycin/PMA-stimulated Jurkat cell group was included as a negative control to provide a baseline for NFAT activation. Subsequent experiments with the PI3K inhibitors duvelisib (DV), TG1011-115, PIK-293 (MedChem Express, HY-17044, HY-10111, and HY-13504, respectively) were performed at 10 µM based on cytotoxicity data (Supplementary Figure S4). For studies investigating whether the effects of DV were due to PI3K inhibition, DV (10 µM) and the GSK3β inhibitor tideglusib (25 μM, Cayman Chemical, 16727) were used together for 24 h, based on concomitant agent cytotoxicity data (Supplementary Figure S4). Similarly, TG1011-115 and PIK-293 (10 µM) were included as PI3K inhibitor controls in combination with tideglusib. The percent of NFAT inhibition (%NFAT Inhibition) was calculated as: %NFAT Inhibition = (Vehicle Signal -Sample Signal)/Vehicle Signal × 100. For studies involving tideglusib and its effect on NFAT inhibition, the change in NFAT inhibition (Δ% NFAT inhibition) was calculated as follows: Δ% NFAT inhibition = (%NFAT inhibition without tideglusib - %NFAT inhibition with tideglusib)/%NFAT inhibition without tideglusib × 100.

NIH-3T3 cells (mouse colorectal fibroblasts) were purchased from American Type Culture Collection (ATCC) to investigate specific NFAT protein inhibition due to their low to negligible endogenous NFAT expression. For culturing NIH-3T3 cells, 10% bovine calf serum was supplemented to DMEM (Dulbecco’s modified Eagle’s medium). To assess the inhibition of specific NFAT proteins, we co-transfected the pGL3-NFAT luciferase plasmid with either the pMIG-hNFATc1/bC or pMIG-hNFATc2 plasmid. pMIG-hNFATc1/bC and pMIG-hNFATc2 were gifts from Ria Baumgrass (Addgene plasmid #s 74049, 74050) (Gabriel et al., 2016). TransIT-X2 (MirusBio Systems) was used for the transfection, and 24 h after the transfection, cells were treated with either duvelisib (DV, 10 μM) or cyclosporin A (CsA, 1 μM) for 24 h. We used a similar method to determine the effects of DV (10 μM) or CsA (1 μM) on constitutively active (CA)-NFATC2, obtained from Addgene (plasmid #11102).

### Western blotting assay

Jurkat cells were lysed using the Nuclear and Cytoplasmic Extraction kit (NE-PER, Pierce Biotechnology, Rockford, IL) to obtain separate cytosolic and nuclear NFAT fractions. The protein concentration of the lysate was determined to be at least 1 μg/μL using the BCA assay (BCA Kit; Pierce Biotechnology). Subsequently, 10 μg of protein was separated by sodium dodecyl sulfate-polyacrylamide gel electrophoresis (SDS-PAGE) using 4%–15% Mini-PROTEAN Precast Protein Gels (Bio-Rad) and transferred to a methanol-activated PVDF membrane at 100 V for 45 min. After blocking the membrane with 5% nonfat milk in Tris-buffered saline containing 0.05% Tween (TBST) for 1 h, it was incubated overnight at 4°C with the following primary antibodies purchased from Cell Signaling Technology: NFATC1 (D15F1, #8032) at 1:1,000, NFATC2 (D43B1, #5861) at 1:5,000, GAPDH (D16H11, #5174) at 1:5,000, Beta-Actin (D6A8, #8457) at 1:5,000, and Histone H3 (D1H2, #4499) at 1:5,000. The membrane was then washed and incubated for 1 h with horseradish peroxidase-conjugated goat anti-rabbit IgG (1:10,000, Cell Signaling Technology, #7074). The membrane was developed using a chemiluminescence detection reagent (Amersham ECL) and exposed to film. Quantification of Western blotting results was performed using ImageJ software. To quantify and calculate the ratio of NFAT expression in nuclear to cytoplasmic extracts, Histone 3 and GAPDH were used to normalize NFAT expression in the nucleus and cytoplasm, respectively.

### Drug affinity responsive target stability (DARTS) assay

To investigate drug-protein interactions in Jurkat cells, the DARTS assay was performed, using a previously described method (Lomenick et al., 2011). Cells were lysed with RIPA buffer containing Halt Protease Inhibitor Cocktail (Pierce Biotechnology) and incubated the resulting cell lysates with duvelisib (1 mM) or a DMSO control. Samples were digested with pronase enzyme (1:1,000 Pronase: protein ratio for 10 min, Roche) and the reaction was quenched with 20X Halt on ice for 10 min. After boiling the samples at 95°C for 5 min, we used Western blotting with specific antibodies to investigate the binding of the drug molecules to NFATC2.

### Calcineurin phosphatase activity assay

The effect of DV (10 μM) or CsA (1 μM) on calcineurin phosphatase activity was assessed using a commercially available assay kit (#AB139461) from Abcam, following the manufacturer’s instructions.

### Development of a murine model of adalimumab immunogenicity

The Institutional Animal Care and Use Committee (IACUC) of the University of Pittsburgh approved all experiments, which were conducted in compliance with the NIH guidelines for the care and use of laboratory animals. Mice were housed on a 12-h light-dark cycle (0700–1900) and had access to normal chow and water *ad libitum*. To create a murine model of adalimumab immunogenicity, we administered two intraperitoneal injections (27G × 1/2″ needle, EXELINT, #26400) of 150 µL adalimumab (10 μg, Humira, Abbott Laboratories; Abbott Park, IL) formulated with 1 mg of aluminum hydroxide adjuvant (Imject Alum; Thermo Scientific, Rockford, IL) to eight-week-old female C57BL/6J mice (Jackson Laboratory, Stock #000664), separated by 7 days, as previously described by our group (Fernandez et al., 2013; Rathod et al., 2018; Rathod et al., 2020).

The following intravenously administered treatment groups (25G × 5/8″ needle, EXELINT, #26046) were administered concomitantly with the adalimumab immunization to investigate strategies for mitigating adalimumab immunogenicity: DV at 22.5 mg/kg (n = 3) or 45 mg/kg (n = 4), MTX at 5 mg/kg (n = 3), and a vehicle control (n = 4). The dosage of DV used in our study is based on previous preclinical investigations demonstrating efficacy and no apparent *in vivo* toxicity (Li et al., 2023). Similarly, the 5 mg/kg dose of MTX was selected based on prior studies investigating its ability to mitigate anti-drug antibody responses in preclinical models (Herskovitz et al., 2017). To ensure proper experimental controls, we included a positive control for the development of adalimumab immunogenicity, where vehicle-treated mice received two adalimumab immunization doses formulated with aluminum hydroxide adjuvant but were not treated with MTX or DV. Additionally, as a negative control for mice that do not develop adalimumab immunogenicity, we used mice that received the aluminum hydroxide adjuvant without adalimumab or treatment with MTX or DV.

### Detection of anti-adalimumab antibodies by ELISA

Ninety-six-well microtiter plates (Corning, United States) were coated with 0.88 μg/mL of adalimumab (AbbVie Inc., United States) in 100 µL phosphate buffered saline (PBS) overnight at 4°C. The plates were blocked with 2% bovine serum albumin (BSA) in 1X PBS for 1.5 h at room temperature (RT). After washing, 100 µL of sample or controls were added (diluted 1:10,000 in blocking buffer) and incubated at RT for 1 h. Positive control was 0.625 μg/mL of anti-adalimumab IgG1 (Thermo Fisher Scientific, #A01956-40) and negative controls were naïve mouse serum samples. 100 μL of horseradish peroxidase (HRP)-conjugated anti-mouse secondary antibody (1.5 μg/mL, Cell Signaling Technology, #7076S) were added and incubated at RT for 1 h. After washing, 100 µL of o-phenylenediamine dihydrochloride (Sigma Aldrich, #P1526-10G) was added and incubated for 30 min in the dark. The reaction was quenched with 100 µL/well of 1 M phosphoric acid, and absorbance at 490 nm and 630 nm were measured. The difference in wavelengths was used to obtain reference values for analysis. To verify antibody neutralization or binding of antibodies to adalimumab, increasing concentrations of adalimumab (100–500 ng/mL) were spiked into serum samples at a dilution of 1:10,000. The purpose of this assay was to measure the reduction in anti-adalimumab antibody levels following the addition of adalimumab, indicated by the decrease in OD values, as adalimumab binds to and neutralizes these antibodies. To compare differences in anti-adalimumab antibody levels by treatment and across the various added adalimumab concentrations, the area under the antibody-concentration curve (AUC) was calculated using the trapezoidal rule, similar to our previous studies (Liu et al., 2012; Fernandez et al., 2015a). Samples with lower antibody titers are expected to show a greater decrease in both antibody levels and AUC compared to samples with higher antibody titers.

### *Ex-vivo* CD4^+^ T cell proliferation assay

96-well plates (Fisherbrand™ Surface Treated Sterile Tissue Culture Plates) were coated with anti-CD3 antibodies optimized for effective T cell proliferation. For murine splenocyte experiments, plates were coated with 1.26 μg/mL of anti-CD3 (clone 145-2C11, BioLegend) and incubated overnight at 4°C, while for human CD4^+^ T cell experiments, plates were coated with 0.5 μg/mL of anti-CD3 (clone UCHT1, BioLegend) and incubated for 2 h at 37°C. Both conditions were optimized to achieve effective T cell proliferation while minimizing cell death. Murine spleens were harvested from euthanized mice, washed in RPMI and single cell suspensions were made through a 40 µM nylon cell strainer, and red blood cells lysed using ACK lysis buffer. Human PBMCs were obtained from Stemcell Technologies (#70025.2). Isolated splenocyte suspensions and human PBMCs were labeled with CFSE or Cell Trace Violet diluted in PBS at ratios of 1:100 and 1:10,000, respectively. After labeling, excess dye was quenched by incubating the cells in 5 mL of complete media for 5 min, followed by centrifugation at 300 x g for 5 min at room temperature. The supernatant was discarded, and the washing process was repeated once to ensure thorough removal of excess dye. After washing, splenocytes or human PBMCs were plated and co-stimulated with anti-CD28 and treated with either MTX (10 µM) or DV (10 µM) to assess their ability to inhibit CD4^+^ T cell proliferation. Following incubation, cells were initially stained with Zombie NIR or Zombie Aqua (Fixable Viability kit, BioLegend) to identify live cells. Subsequently, surface markers were stained at 4°C for 25 min in the dark with the appropriate antibodies for each cell type: human PBMCs were stained with anti-human CD45-FITC (clone HI30, BioLegend), anti-human CD4-APC (clone RPA-T4, BioLegend), and anti-human TCRβ APC Cy7 (clone IP26, BioLegend), while mouse splenocytes were stained with anti-mouse CD4-APC (clone RM4-4, BioLegend), anti-mouse CD8-PE (clone 53-6.7, BioLegend), and anti-mouse TCRβ PE Cy7 (clone H57-597, BioLegend). The gating strategy sequentially selected live cells (Zombie NIR or Zombie Aqua negative), CD45^+^ leukocytes, TCRβ^+^ T cells, and CD4^+^ T cells. Doublet discrimination was performed by gating single cells based on FSC-H versus FSC-A to exclude aggregated cells. The flow cytometry results were analyzed using FlowJo v10.10 (BD Bioscience), and the triplicate data were concatenated into one sample per treatment group using the concatenate function.

### *Ex-vivo* B cell antibody secretion assay

To induce the differentiation of B cells into antibody-secreting cells and assess the effect of inhibitors on antibody production, splenocytes were added at 250,000 cells/well to 96-well plates with anti-CD40 (eBio, 1 mg/mL), recombinant mouse IL-4 (BioLegend, 200 μg/mL), and recombinant mouse IL-21 (BioLegend, 100 μg/mL) and incubated at 37°C for 3 days, following a previously described method (Franke et al., 2020). The effect of DV (10 µM) or MTX (10 µM) on antibody secretion was determined by measuring total IgG secretion using the anti-mouse total IgG ELISA kit (Thermo Scientific, Rockford, IL).

### Electrophoretic mobility shift assay (EMSA)

NIH-3T3 cells were transfected with pMIG-hNFATc2 plasmid using TransIT-X2 (MirusBio Systems) (Gabriel et al., 2016). Transfected cells were stimulated with phorbol 12-myristate 13-acetate (PMA, 50 ng/mL) and ionomycin (3 μg/mL) for 2 h, and the nuclear lysate was extracted using the nuclear cytoplasmic extract kit (NE-PER, Pierce Biotechnology, Rockford, IL). The BCA Assay was used to determine the concentration of nuclear NFAT, and samples were diluted to 5 μg/μL. Samples were prepared using the Odyssey EMSA kit (LI-COR) as per manufacturer instructions. Labeled hARRE-2 oligonucleotides (5′ CAA AGA GGA AAA ACT GTT TCA TAC AG 3′, IDT) or TNFα oligonucleotides (5′ GAG CTC ATG GGT TTC TCC ACC 3′, IDT) were diluted in IDEx water and added to samples. We used an NFATC2 antibody (cell signaling) to produce a supershift in the observed NFATC2 band during the EMSA procedure. Samples were incubated with 1 μM, 100 μM, and 1 mM of duvelisib. After 30 min of incubation, 10x orange loading dye was added, and the samples were loaded on agarose gel and electrophoresed for 50 min. The exposure time was 3.5 min.

### Statistical analysis

Statistical analysis was performed using GraphPad Prism 9 statistical software. All data were analyzed using a two-tailed, unpaired Student’s t-test and expressed as mean ± standard deviation. For comparisons involving more than two groups, ANOVA was performed followed by *post hoc* Dunnett’s multiple comparisons test. A *P*-value of ≤0.05 was considered significant, denoted as *P < 0.05, **P < 0.01, ***P < 0.001, and ****P < 0.0001.

## Results

### Duvelisib is a novel NFAT inhibitor

Our previous studies indicate that inhibition of NFATC2 attenuates drug-induced immunogenicity (Fernandez et al., 2015b; Rathod et al., 2020). The objective of this study was to identify novel pharmacological inhibitors of NFAT and evaluate their effectiveness in protecting against immunogenic responses to adalimumab, a TNFα inhibitor. We therefore performed a computational analysis to identify potential small molecules that may bind directly or interact with NFATC2. Simulation of the DNA-bound structure of NFATC2 identified five transient pockets. Simulating NFATC2 without DNA identified four N-terminal domain pockets with an iso-value of 0.68. However, two of these pockets were deprioritized for further analysis as they were shallow and had limited interaction potential. Simulating NFATC2 with DNA identified an additional pocket at the DNA-binding interface with an iso-value of 0.82. GNINA was used for probe docking three highly druggable snapshots, consisting of three pockets in the N-terminal domain and one in the DNA-binding interface. Using the best-docked poses of the probe molecules, pharmacophores were generated for each snapshot of every pocket. Subsequently, the MolPort database was screened for molecules that matched these pharmacophores, and GNINA was used to prioritize molecules based on their scores resulting in a selection of 31 commercially available molecules.

We screened these compounds for their NFAT inhibitory effects using Jurkat-Lucia NFAT cells, with CsA and FK506 as controls (Supplementary Table S1). Five potential NFAT inhibitors were identified, including duvelisib (DV), a clinically available PI3K inhibitor which was predicted to bind in the DNA binding pocket of NFATC2. All 5 identified compounds exhibited high NFAT inhibition at 10 µM concentration (>70%) which was found to be comparable to CsA (Supplementary Table S1). Since DV is an approved agent for treating chronic lymphocytic leukemia with known and suitable preclinical and clinical ADME/PK parameters, we chose to focus our studies on this agent (Rodrigues et al., 2019).

**FIGURE 1:**
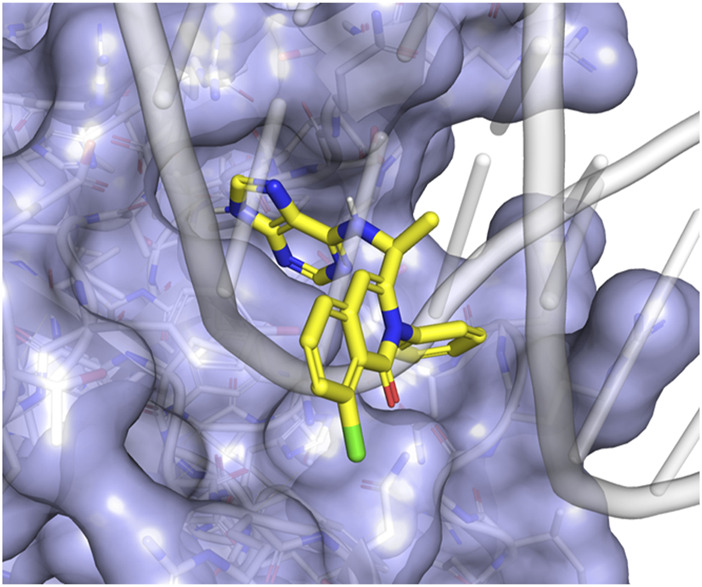
Duvelisib is predicted to bind to NFATC2 at the DNA-binding interface. (A) The putative binding mode of duvelisib (yellow sticks) places the purine motif in a pocket adjacent to the DNA binding site. This placement allows the remaining part of the molecule to block the binding of the DNA backbone (grey cartoon) to NFATC2 (blue surface).

Our computational analyses suggest that DV would bind to the pocket identified in the DNA-binding interface (Figure 1) with a Vina affinity of 7.16, Crossdock_Default2018 affinity of 6.29, and a Dense affinity of 5.08. DV is predicted to form hydrogen bonds with ARG421, ASN523, and the backbones of ALA422 and CYS569, and have an aromatic interaction with TYR424. DV contains a purine motif, but its predicted binding mode situates the purine in a different position compared to the purines found in the bound DNA. The binding pocket where DV attaches does not have direct interactions with DNA during the simulation. Nevertheless, it is situated in close proximity to the DNA backbone binding interface. We predict that the binding of DV to this NFATC2 pocket would likely interfere with its ability to bind to the DNA backbone, thereby disrupting its function as a transcription factor.

**FIGURE 2:**
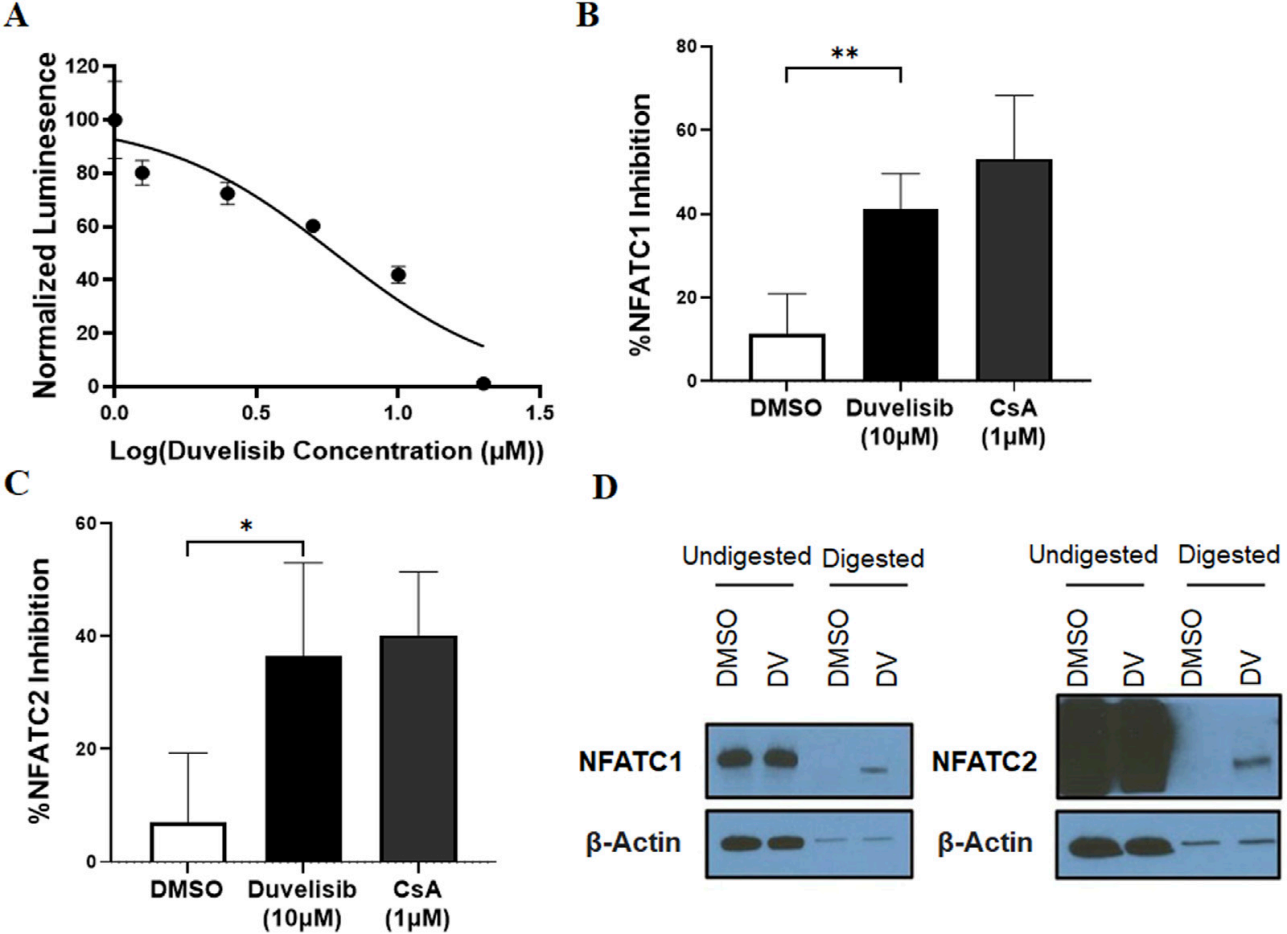
Duvelisib (DV) is a novel NFAT inhibitor. **(A)** DV dose-dependently inhibits NFAT activity in PMA/ionomycin-stimulated Jurkat luciferase reporter cells. DV (10 µM) significantly inhibits **(B)** NFATC1 or **(C)** NFATC2 activity in NIH-3T3 cells with similar potency as cyclosporin A (CsA, 1 µM). **(D)** DARTS analysis, which measures protein stability changes upon drug binding, suggests a direct interaction between DV and NFATC1/2, but not the control protein β-actin. * and ** indicate *P* < 0.05 and 0.01, respectively.

To validate our initial screening, we conducted dose-response experiments using Jurkat-Lucia NFAT cells and determined that DV inhibited NFAT activation with an IC50 of 5.975 µM (Figure 2A). However, since NFAT family members share sequence homology and multiple members can bind the IL2 promoter, we then evaluated the selectivity of DV NFAT inhibition. Due to the high expression of NFATC2 and NFATC1 in T (Monticelli and Rao, 2002; Lee et al., 2018) and B cells (Vaeth and Feske, 2018), and the largely conserved DNA binding sites of NFATC2 and NFATC1, we investigated whether DV could inhibit both NFATC2 and NFATC1. To do so, we transfected NIH-3T3 cells with NFATC1 or NFATC2 plasmids and then treated them with DV prior to stimulation with ionomycin and PMA. Our results demonstrate that DV significantly inhibited both NFATC2 and NFATC1 (Figures 2B, C). This dual inhibition is essential for achieving more potent protection against drug-induced immunogenicity (Rathod et al., 2020).

To investigate our hypothesis that DV directly binds to NFATC2/1, we performed a Drug Affinity Responsive Target Stability (DARTS) assay using Jurkat cell lysate treated with DV. DARTS is a reliable method for identifying potential protein targets of small molecules (Pai et al., 2015). This assay detects alterations in the biophysical characteristics of protein targets caused by the binding of small molecules, such as changes in proteolytic degradation (Hwang et al., 2020), although higher concentrations (≥10-fold higher than those needed for biological effects (Lomenick et al., 2011; Huang et al., 2021)) are required. Our findings are consistent with the hypothesis that DV binds to and inhibits NFATC2/1, where we observed that DV (1 mM) reduced the protease digestion of NFATC2/1 relative to our negative control, β-actin, which was similarly digested regardless of DV treatment (Figure 2D). To confirm these findings, we developed a Cellular Thermal Shift Assay (CETSA) to orthogonally evaluate the interaction between NFAT and DV. The CETSA results are consistent with our DARTS data, demonstrating that DV (1 mM) substantially protects NFATC2 from heat-induced degradation and modestly increases the thermal stability of NFATC1 (Supplementary Figure S5). Importantly, the effect of DV is specific to NFAT, as it does not provide any protective effect for β-actin. Our results support the conclusion that DV is a novel inhibitor of NFATC2/1 activation.

### Duvelisib inhibits the transcriptional activity of NFAT without preventing its nuclear translocation

**FIGURE 3:**
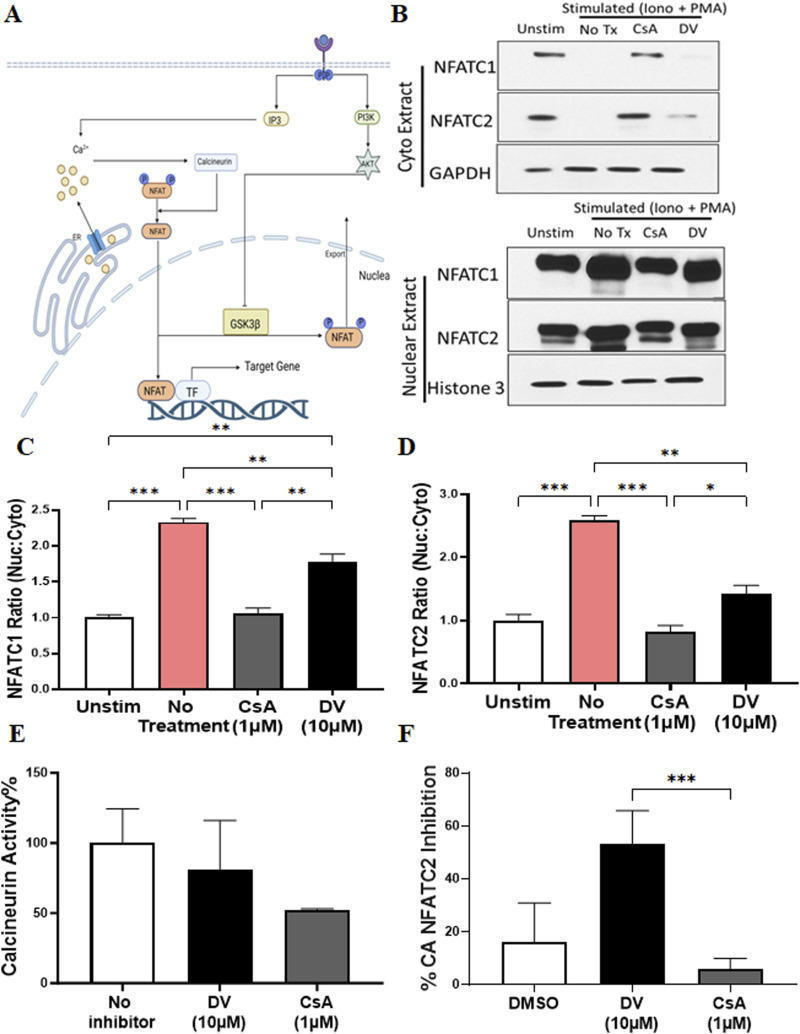
Duvelisib (DV) inhibits NFAT transcriptional activity without affecting its nuclear translocation. **(A)** Schematic representation of the calcium-calcineurin-NFAT signaling pathway, highlighting potential targets affected by DV. **(B–D)** Compared to CsA and non-stimulated Jurkat cells, DV (10 µM) significantly sustains elevated levels of NFATC1/2 within the nucleus, as shown by Western blot analysis. Bar graphs show the quantified NFATC2/1 ratio (nuclear: cytoplasmic) for each treatment. **(E)** DV (10 µM) does not inhibit calcineurin activity. **(F)** DV (10 µM) but not CsA (1 µM) inhibits the transcriptional activity of constitutively active NFATC2. *, **, *** indicate *P* < 0.05, 0.01, and 0.001, respectively.

There are several mechanisms by which a compound can inhibit the transcriptional activity of NFATs, including blocking intracellular calcium release, inhibiting calcineurin phosphatase activity, hindering calcineurin-NFAT interaction, restricting NFAT nuclear localization, and preventing NFAT-transcription factor/DNA binding (Figure 3A). To elucidate the mechanism by which DV attenuates NFAT activation after ionomycin and PMA stimulation, we investigated whether DV (10 µM) reduces NFATC2/1 nuclear translocation, similar to CsA, which inhibits NFAT nuclear localization by blocking calcineurin-mediated NFAT dephosphorylation. Our results indicate that CsA significantly reduces the nuclear levels of NFATC2/1 proteins, similar to those observed in non-stimulated Jurkat cells (Figures 3B–D). In contrast, DV maintains higher nuclear NFATC1/2 compared to CsA and non-stimulated Jurkat cells (Figures 3B–D). These findings suggest that the mechanism by which DV inhibits NFATC1/2 transcriptional activity likely does not involve blocking nuclear translocation.

Since the nuclear localization of NFAT after dephosphorylation requires calmodulin-mediated calcineurin activation (Figure 3A), we investigated whether DV inhibits the phosphatase activity of calcineurin. Consistent with our results from Western blotting of nuclear and cytoplasmic fractions, our data show that DV (10 µM) does not attenuate calcineurin activity, whereas CsA (1 µM) reduces the enzymatic activity of calcineurin as expected (Figure 3E). Our results suggest that DV does not impact calcium mobilization, calcineurin activation via calmodulin, or the phosphatase activity of calcineurin.

To validate that DV inhibits the transcriptional activity of nuclear NFAT, we used a constitutively active NFATC2 (CA-NFATC2) containing multiple serine-to-alanine substitutions in its regulatory domain, maintaining NFATC2 dephosphorylated and within the nucleus (Monticelli and Rao, 2002). Since the transcriptional activity of CA-NFATC2 is independent of calcineurin phosphatase enzyme activity, we used CsA as a control, which should not affect the transcriptional activity of CA-NFATC2. Consistent with our Western blotting results indicating that DV likely does not inhibit NFAT by blocking its nuclear translocation, our findings suggest that DV treatment, but not CsA, potently attenuates the transcriptional activity of CA-NFATC2 (Figure 3F). Altogether, our results provide compelling evidence that DV inhibits the transcriptional activity of nuclear NFAT through a mechanism of action that is independent of calcineurin.

### The NFAT inhibitory effects of duvelisib are independent of PI3K suppression

**FIGURE 4:**
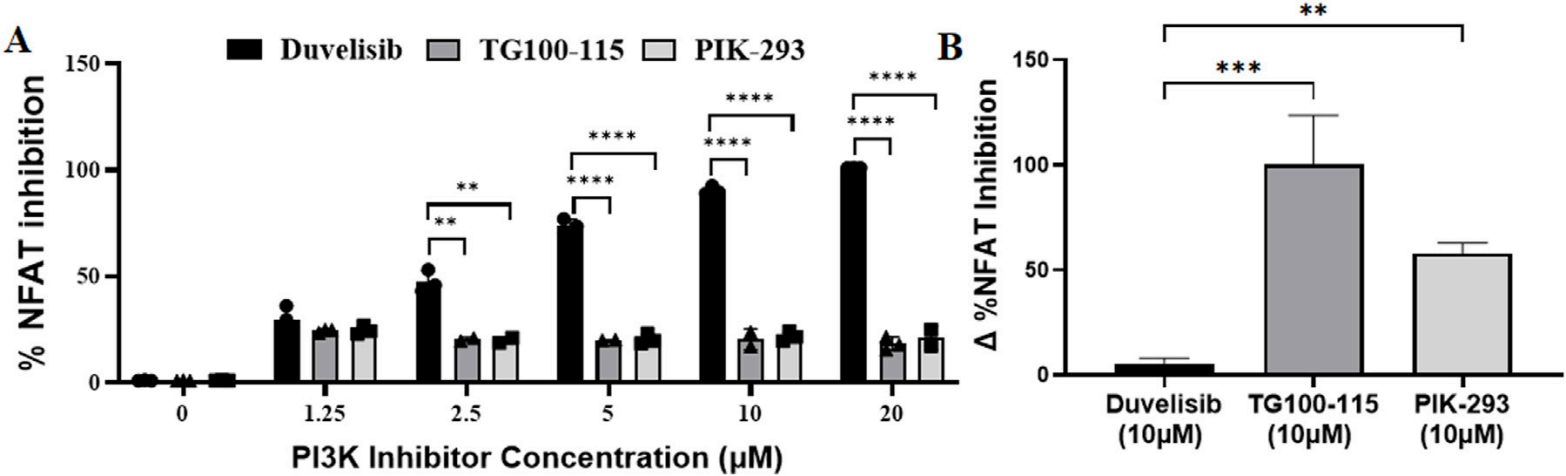
The effects of DV on NFAT are independent of PI3K suppression. To assess whether the inhibition of NFAT activation by DV was due to its PI3K inhibitory properties, we evaluated the effect of other PI3K inhibitors on NFAT activation. **(A)** Unlike DV, the PI3K inhibitors TG100-115 and PIK-293 failed to potently inhibit NFAT transcriptional activity in PMA/ionomycin-stimulated Jurkat luciferase reporter cells (1.25–20 μM). **(B)** Because PI3K negatively regulates GSK3, which can regulate NFAT activation through phosphorylation, we also assessed whether the effects of DV were due to GSK3β activation using the GSK3β inhibitor, tideglusib. Tideglusib (25 µM) did not affect the NFAT inhibitory activity of DV (10 µM) in Jurkat cells. In contrast, while TG100-115 and PI3K inhibited NFAT activation by less than 25%, this effect was strongly attenuated by GSK3β inhibition with tideglusib. The Δ% NFAT inhibition represents the change in NFAT inhibition with and without tideglusib treatment. **, ***, and **** indicate *P* < 0.01, 0.001, and 0.0001 respectively.

Previous studies have indicated that the PI3K-Akt signaling pathway promotes NFAT-mediated transcriptional activity by reducing the activity of GSK3β, which phosphorylates and deactivates NFATs (Kim et al., 2014). Given that DV is an inhibitor of PI3Kγδ (Peluso et al., 2014), our next objective was to investigate whether DV inhibited NFAT activation by suppressing PI3K activity (Figure 4A). To test the hypothesis that PI3K inhibition is necessary for the mechanism of action of DV, we evaluated the impact of pharmacological PI3K inhibition on the transcriptional activity of NFAT using Jurkat-Lucia NFAT cells and two PI3Kγδ inhibitors, TG100-115 and PIK-293 (Supplementary Table S2). Our experiments with DV and the two PI3Kγδ inhibitors showed that TG100-115 and PIK-293 only slightly inhibited NFAT transcriptional activity, while DV significantly attenuated its activation at concentrations of 2.5, 5, 10, and 20 µM compared to TG100-115 and PIK-293 (*P* < 0.01, Figure 4A). Since PI3K inhibitors may inhibit NFAT activation by blocking Akt-mediated inactivation of GSK3β, leading to more dephosphorylated NFAT and increased nuclear NFAT (Figure 3A), we investigated whether the effect of DV on NFAT activation could be counteracted by inhibiting GSK3β activity with tideglusib. Our findings indicate that tideglusib effectively prevented the slight inhibition of NFAT by TG100-115 and PIK-293 we observed, whereas NFAT inhibition by DV remained unaffected by the pharmacological inhibition of GSK3β (Figure 4B). Overall, our results support that the effect of DV on NFAT inhibition may not depend on its PI3K inhibition properties.

### Duvelisib disrupts NFAT binding to DNA

**FIGURE 5:**
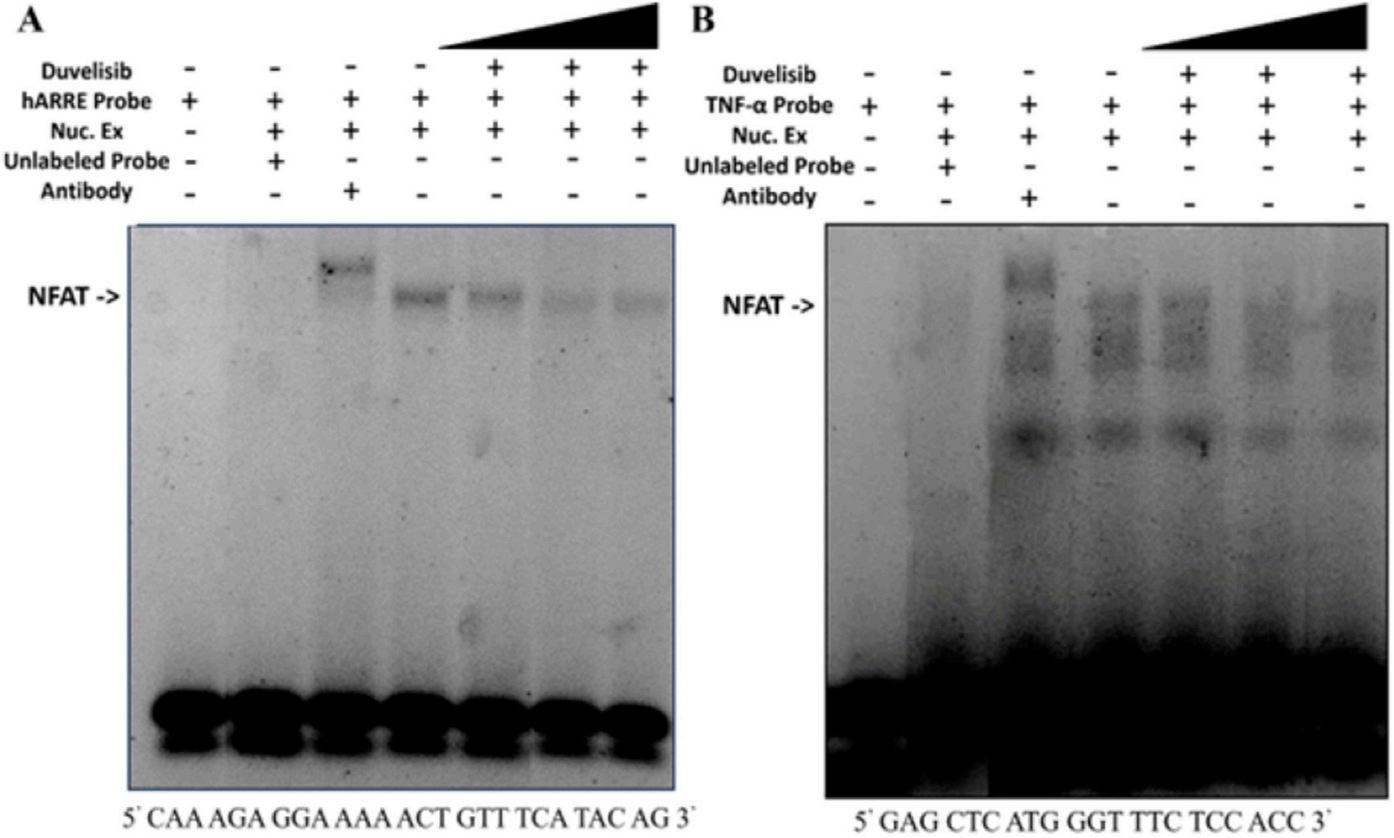
DV attenuates NFAT binding to DNA. Nuclear extracts (5 µg) from NIH-3T3 cells transfected with NFATC2 were incubated with IRDye-labeled oligonucleotides (100 nM) corresponding to **(A)** the antigen-receptor response element-2 (ARRE-2) site of the Il2 promoter, which contains a consensus binding site for NFAT, or **(B)** the κ3 element of the TNF promoter, which can bind NFAT as a dimer. The resulting band shifts indicate binding between nuclear NFATC1/2 and the probe (lane 1 vs. 4). Addition of DV (1 μM, 100 μM, or 1 mM; lanes 5–7), anti-NFATC2 antibody (lane 3), or unlabeled cold probe (lane 2) disrupted the probe-NFATC1/2 interaction.

Based on computational simulations suggesting that DV may be able to bind NFAT and our cumulative experimental results indicating that DV disrupts nuclear NFAT activity, we postulated that DV inhibits the transcriptional activity of NFAT by disrupting its interaction with DNA. NFATC1/2 share a conserved DNA binding domain that recognizes the consensus sequence (A/TGGAAA) (Rao et al., 1997). To investigate whether DV affects this binding interaction, we performed an electrophoretic mobility shift assay (EMSA). This assay involved using double-stranded oligonucleotides targeting either the antigen-receptor response element-2 (ARRE-2) in the Il2 promoter or the κ3 element in the TNF promoter. At the ARRE-2, IL-2 transcription requires cooperation of NFAT with AP-1 (Macian et al., 2001), whereas the initiation of TNF production is dependent on the formation of NFAT dimers (Macian et al., 2001). Our results show that incubating nuclear extracts from transfected NIH-3T3 cells with either of the two labeled oligonucleotide probes results in a band shift, indicative of NFAT binding (Figures 5A, B). This interaction is disrupted by the addition of an unlabeled, cold probe (10X) of identical sequence, and a supershift is observed upon the addition of an anti-NFATC2 antibody, confirming the NFATC2-DNA interaction (Figures 5A, B). Similar to the effect of excess unlabeled probe, increasing concentrations of DV (1 μM, 100 μM, 1 mM) leads to a concentration-dependent decrease in the interaction between the probe and NFATC2 (Figures 5A, B). Our results support that DV attenuates the transcriptional activity of NFAT by interfering with its DNA binding capabilities.

### Duvelisib protects mice from adalimumab-mediated immunogenicity

**FIGURE 6:**
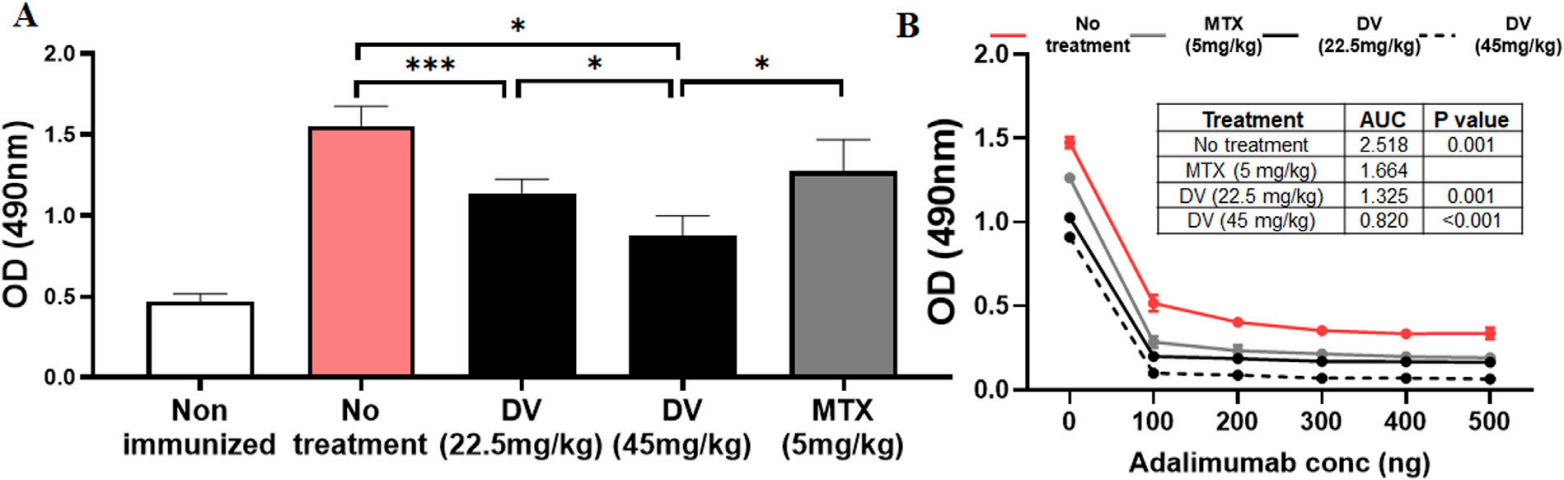
DV protects mice from adalimumab-induced immunogenicity. **(A)** Treating mice with DV (22.5 or 45 mg/kg) or MTX (5 mg/kg) reduced serum anti-adalimumab antibody levels compared to non-treated controls, as measured by ELISA. **(B)** Adding adalimumab to the serum of immunized mice resulted in a decrease in anti-adalimumab antibody optical density (OD), which was quantified by estimating the antibody AUC. The table within the figure provides statistical comparisons of the AUC values, showing that antibodies generated during immunization bind and neutralize adalimumab. In contrast, treatment with DV or MTX resulted in a greater reduction in antibody levels and AUC upon adalimumab addition, with DV treatment at 45 mg/kg producing significantly lower antibody levels compared to the other treatments. *, **, and *** indicate *P* < 0.05, 0.01, and 0.001, respectively.

Our study has shown that DV has the potential to significantly inhibit the activation of NFAT at clinically relevant concentrations (Flinn et al., 2018). Given our previous research on the role of NFAT in drug-induced immunogenicity, we aimed to investigate whether DV could mitigate *in vivo* immunogenicity to adalimumab in mice. To accomplish this, we developed a murine model of adalimumab immunogenicity based on a previously established model we created (Fernandez et al., 2015a). In this model, mice were immunized with alum-formulated adalimumab on day 0 and day 14 of the protocol, followed by blood sample collection on day 24 to measure anti-adalimumab antibodies. Our results revealed that this approach effectively induced high levels of anti-adalimumab IgG antibodies that bind to and neutralize adalimumab in a concentration-dependent manner (Figures 6A, B).

After establishing a murine model that recapitulated clinical adalimumab immunogenicity, we sought to determine whether concurrent administration of DV or MTX during immunizations could inhibit the development of immunogenicity to adalimumab (Figure 6A). We used MTX as a positive control because it is known to suppress drug-induced immunogenicity (Krieckaert et al., 2012; Vogelzang et al., 2015). Our model recapitulated the expected clinical effect of MTX on adalimumab immunogenicity (Krieckaert et al., 2012), where pretreatment with clinically relevant doses of MTX attenuated the development of adalimumab immunogenicity (Lucas et al., 2019). Moreover, our results showed that DV provided dose-dependent protection against the development of anti-adalimumab antibodies (Figure 6A), including the formation of neutralizing antibodies (Figure 6B, antibody AUC). Interestingly, we observed that a dose of 45 mg/kg of DV demonstrated greater efficacy in mitigating adalimumab immunogenicity compared to MTX treatment (Figures 6A, B), with no mice at any DV dose showing overt signs of toxicity. Collectively, our findings suggest that DV can significantly suppress anti-adalimumab antibodies and provide similar protection compared to MTX.

### Duvelisib suppresses CD4^+^ T cell proliferation and B cell antibody secretion

**FIGURE 7:**
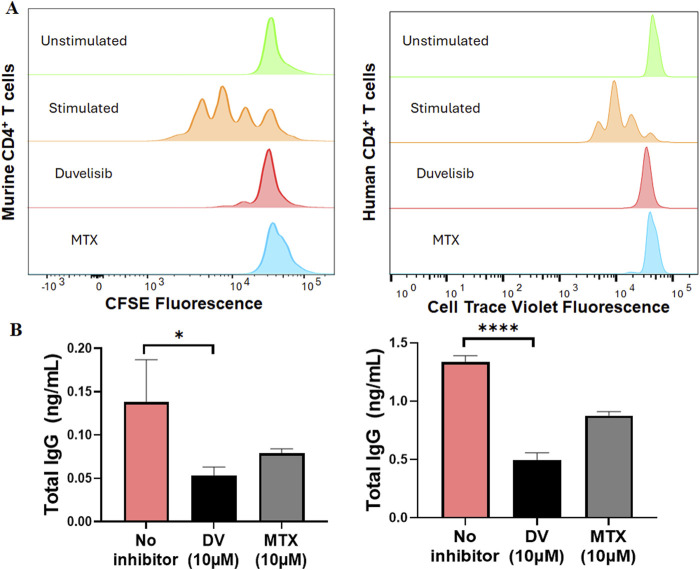
DV suppresses CD4^+^ T cell proliferation and B cell antibody secretion. **(A)** DV (10 µM) and MTX (10 µM) attenuate CD4^+^ T cell proliferation in mouse splenocytes and human PBMCs stimulated with anti-CD3 and anti-CD28 antibodies. **(B)** DV (10 µM) and MTX (10 µM) inhibit B cell antibody secretion induced by anti-CD40, recombinant mouse IL-4, and recombinant mouse IL-21 stimulation in both mouse splenocytes and human PBMCs. *, **, ***, and **** indicate *P* < 0.05, 0.01, 0.001, and 0.0001, respectively.

Building upon previous research indicating that the loss of NFATC2/1 can disrupt cytokine secretion and compromise the activation and function of T and B cells (Peng et al., 2001; Bhattacharyya et al., 2011), we posited that DV may impact anti-adalimumab immunogenicity by inhibiting NFAT in CD4^+^ T and/or B cells. To test this hypothesis, we stimulated splenocytes or human PBMCs with anti-CD3 and anti-CD28 antibodies, with and without DV (10 µM) or MTX (10 µM), and measured CD4^+^ T cell proliferation by labeling cells with CFSE or cell trace violet and monitoring fluorescence halving of daughter cells using flow cytometry. Our data show that DV significantly inhibited CD4^+^ T cell proliferation and division of daughter cells from both murine splenocytes and human PBMCs (Figure 7A). Consistent with prior investigations indicating that MTX suppresses T cell proliferation (Fairbanks et al., 1999), we show that MTX inhibited CD4^+^ T cell proliferation to a degree comparable to unstimulated and DV-treated cells (Figure 7A).

To investigate how DV affects antibody-secreting cells, we differentiated B cells from splenocytes or human PBMCs using a similar method to that described previously (Franke et al., 2020). Using anti-CD40, IL-4, and IL-21 to differentiate B cells, we were able to induce the secretion of IgG antibodies, which we measured by ELISA (Figure 7B). Additionally, we show that treating the cells with DV (10 µM) significantly reduced the concentration of IgG antibodies, indicating that DV has a potent effect on suppressing antibody secretion (Figure 7B). Consistent with previous studies (Bartelds et al., 2011; Krieckaert et al., 2012; Vogelzang et al., 2015), we also show that MTX (10 µM) reduces antibody IgG levels to a similar extent as DV. Our collective findings support that DV protects against the development of *in vivo* adalimumab immunogenicity by suppressing CD4^+^ T cell proliferation and antibody secretion, likely through its ability to inhibit NFAT signaling.

## Discussion

The purpose of this study was to identify a novel NFAT inhibitor that can effectively mitigate drug-induced immunogenicity. Our computational analysis, combined with experiments using Jurkat-Lucia NFAT cells, led us to discover DV as a potent NFAT inhibitor (Figure 1). Dose-response experiments with DV demonstrated significant NFAT inhibition, with an IC50 of 5.975 µM, which falls within clinically feasible duvelisib plasma levels of up to 7.9 µM (Flinn et al., 2018). Because our past studies support that inhibition of NFATC2/1 better protects from drug-induced immunogenicity (Rathod et al., 2020), we evaluated the selectivity of NFAT inhibition by DV. Our results demonstrated that DV inhibited both NFATC2/1 isoforms (Figures 2B, C), and our DARTS and CETSA results showed that DV protects the proteins from proteolytic and thermal degradation (Figure 2D; Supplementary Figure S5). Our investigation into the mechanism of action of DV suggests that it can directly bind to NFAT, disrupting its ability to bind DNA and induce target gene expression. These findings are supported by our results, showing that DV did not reduce the amount of nuclear NFATC2/1 to a similar extent as CsA (Figures 3B–D) or attenuate calcineurin phosphatase activity (Figure 3E). However, DV disrupted the binding of NFAT to its consensus sequence (Figures 5A, B).

We established a murine model of adalimumab immunogenicity to investigate the efficacy of DV in diminishing this immunogenic response. Our model recapitulates key clinical features of adalimumab immunogenicity, such as the formation of high levels of anti-adalimumab IgG antibodies and the effectiveness of MTX in preventing immunogenicity (Figure 6A). Using our model, we demonstrate that DV (22.5 and 45 mg/kg) protects against the development of adalimumab immunogenicity to a similar or more effective extent as MTX (5 mg/kg, Figures 6A, B). The studies we performed using human and murine T and B cells indicate that the mechanism of protection provided by DV may be due to the combined inhibitory effect of DV on CD4^+^ T cell proliferation and B cell antibody secretion (Figures 7A, B). Collectively, our results suggest that NFAT is a pharmacological target for preventing drug-induced immunogenicity and show that it may be targeted directly with small molecules.

Our previous studies have shown that inhibiting NFAT activation using the 11R-VIVIT peptide strongly protects against asparaginase-induced immunogenicity (Rathod et al., 2020). However, due to the limitations of peptide therapeutics, our study aimed to identify a novel small molecule inhibitor of NFAT. We found that DV, a clinically available small molecule, was able to attenuate NFAT activation, while other PI3K inhibitors were not. Nevertheless, our study using the GSK3β inhibitor tideglusib and PI3K inhibitors TG100-115 and PIK-293 lacked controls confirming target inhibition, despite using concentrations often >100-fold their IC50 values (Supplementary Table S2). Therefore, although our results show that DV significantly reduces NFAT activation, further studies, such as genetic inhibition experiments, are needed to clarify the role of PI3K in modulating the effects of DV on NFAT inhibition. Because PI3K inhibition has been shown to attenuate inflammation (Flinn et al., 2018), we cannot exclude the possibility that PI3K inhibition by DV contributes to the observed *in vivo* protection against adalimumab immunogenicity.

Our study aimed to identify tool molecules that can be further developed to reduce immune responses and enhance the therapeutic efficacy of TNFα inhibitors. Our results indicate that DV reduces *ex vivo* T cell proliferation, *ex vivo* B cell antibody secretion, and *in vivo* anti-adalimumab antibody levels. While our findings demonstrate that DV inhibits NFAT and protects against TNFi immunogenicity, they do not suggest that DV is an adequate alternative to MTX in RA therapy. Instead, DV is a dual inhibitor of PI3K-δ and PI3K-γ, FDA-approved for refractory chronic lymphocytic leukemia or small lymphocytic lymphoma (Blair, 2018). However, like other PI3K inhibitors, DV carries safety concerns that have resulted in a black box warning (Skånland and Brown, 2023). Neutropenia is a common adverse effect of DV, associated with an increased risk of death and other serious side effects (Skånland and Brown, 2023). Whether these toxicities arise from on-target effects of PI3K inhibition, off-target effects, or its NFAT inhibition properties remains unclear. Nevertheless, our findings highlight the potential of NFAT inhibition as a mechanism to reduce biologic immunogenicity. Importantly, previous studies, including a 4-week preclinical study in rats with the peptide inhibitor 11R-VIVIT, suggest that global inhibition of NFAT transcriptional activity is safe, as it did not cause liver or renal toxicity and provided protection against cardiovascular disease (Kuriyama et al., 2006). These findings support the feasibility of developing safer, more selective NFAT inhibitors. Future studies should focus on designing NFAT inhibitors that avoid PI3K inhibition and prioritize safety for clinical application.

While our results indicate that DV can reduce *ex vivo* T cell proliferation, *ex vivo* B cell antibody secretion, and *in vivo* anti-adalimumab antibody levels, residual lymphocytes may still retain their effector functions. To address this, future experiments will assess the impact of pharmacological NFAT inhibition on activated T cells by examining activation markers such as CD69 (Pierzchalski et al., 2023). An additional limitation of our study is related to the DARTS and CETSA results that suggest DV can directly interact with NFATC2. Specifically, our study used a single concentration of 1 mM DV to demonstrate that DV protects NFATC2 from proteolytic and thermal degradation, with no effect on β-actin. This concentration was chosen based on preliminary method development to ensure reliable and reproducible results and is consistent with concentrations used in prior DARTS studies (Nishi et al., 2023). While we demonstrated temperature-dependent protection of NFATC2 from heat-induced degradation by DV, additional experiments, such as dose-response analyses, are needed to validate and further support these findings. Nevertheless, small molecule NFAT inhibitors that specifically mitigate nuclear NFAT activity, rather than targeting calcineurin like CsA or relying on potentially cytotoxic antimetabolites, offer a more targeted approach to suppressing T cell activation and reducing immunogenicity compared to traditional immunosuppressive agents. Our future studies will focus on assessing the co-crystal structure of DV with NFATC2 to elucidate the mechanisms of their interaction and to inform future structure-activity relationship studies. Despite the PI3K inhibition properties of DV, our findings demonstrate the feasibility of targeting NFAT pharmacologically to protect against TNFi immunogenicity. Building on these results, future efforts will prioritize the rational design of DV analogs that specifically inhibit NFAT while eliminating PI3K inhibition properties, thereby improving specificity and minimizing potential non-NFAT-related toxicities. This approach has the potential to optimize the safety and efficacy of NFAT inhibitors for clinical translation, while addressing the limitations of DV.

In summary, our computational approach identified small molecules predicted to bind to the DNA pocket of NFATC2. We discovered that DV can act as a NFATC2/1 inhibitor and can protect against adalimumab immunogenicity in a dose-dependent manner by suppressing CD4^+^ T cell proliferation and B cell antibody secretion. Interestingly, DV achieves this by attenuating the transcriptional activity of NFAT rather than by inhibiting calcineurin-mediated NFAT dephosphorylation, which is a distinct mechanism from other strategies to attenuate NFAT activation. Our future directions are to build on these findings by developing novel, safe NFAT inhibitors that can disrupt the interaction between these transcription factors and their target genes, ultimately paving the way for potential clinical applications.

## Data Availability

The raw data supporting the conclusions of this article will be made available by the authors, without undue reservation.
