# Supplementary material for: Duvelisib is a novel NFAT inhibitor that mitigates adalimumab-induced immunogenicity
Source: Front Pharmacol. 2025 Jan 9;15:1397995. doi: 10.3389/fphar.2024.1397995 (PMC11754251; doi:10.3389/fphar.2024.1397995)
Supplement: Supplementary file 1 [file DataSheet1.pdf]

SUPPLEMENTARY MATERIAL

Duvelisib is a novel NFAT inhibitor that mitigates adalimumab-induced immunogenicity.

Aboli Bhingarkar,[1*] Yuyin Wang,[1*] Keito Hoshitsuki,[1] Katherine Marie Eichinger,[1,2] Sanjay Rathod,[1] Yin Zhu,[1] He Lyu,[1] Andrew T. McNutt,[3] Larry W Moreland,[4] Lee McDermott,[5] David R. Koes,[3] and Christian A. Fernandez[1]

[1]Center for Pharmacogenetics and Department of Pharmaceutical Sciences, University of Pittsburgh School of Pharmacy, Pittsburgh, PA 15261, USA

[2]Duo Oncology, Pittsburgh, PA 15207, USA

[3]Department of Computational and Systems Biology, School of Medicine, University of Pittsburgh, Pittsburgh, PA 15261, USA

[4]Division of Rheumatology, School of Medicine, University of Colorado, Aurora, CO, USA

[5]Department of Pharmaceutical Sciences, University of Pittsburgh School of Pharmacy, Pittsburgh, PA 15261, USA

*Both authors contributed equally to this manuscript.

Running Title:

NFAT inhibition mitigates adalimumab-induced immunogenicity.

Corresponding Author:
Christian A. Fernandez
Center for Pharmacogenetics
Department of Pharmaceutical Sciences
School of Pharmacy
335 Sutherland Drive
Pittsburgh, PA 15261
Phone: (412) 383-8108
chf63@pitt.edu

SUPPLEMENTAL METHODS

Cellular Thermal Shift Assay (CETSA) for assessing duvelisib binding to NFAT

The binding of duvelisib to NFATC2 and NFATC1 was analyzed using a cellular thermal shift assay (CETSA), following methods similar to those in previous studies (Wang et al., 2018;Dayalan Naidu et al., 2022). In this assay, the binding of a small molecule to its target protein stabilizes the protein, protecting it from heat-induced degradation. First, 2×10^7 Jurkat cells were washed twice with PBS and resuspended in 1X Halt Protease and Phosphatase Inhibitor Single-Use Cocktail (Thermo Scientific, Rockford, IL) in PBS. The cells were then vortexed, snap-frozen in liquid nitrogen for 2 minutes, and thawed in a 25°C water bath. This freeze-thaw cycle was repeated four times to prepare the cell lysates. The lysate was extracted by centrifugation at 17,000xg for 40 minutes at 4 °C. Next, the cell lysate was then treated with 1 mM duvelisib or vehicle (2.5% DMSO) for 30 minutes at 4 °C. The samples were heated at temperatures ranging from 40-85 °C followed by cooling for 5 minutes at room temperature (S1000 Thermal Cycler, Bio-Rad). The effect of duvelisib on NFATC2 and NFATC1 stabilization was determined by measuring protein levels through Western blot analysis.

SUPPLEMENTAL TABLES

Supplemental Table 1

TABLE 1. List of compounds with % NFAT inhibition

Molport ID	Vendor	% NFAT inhibitor	Structure
Molport-023-220-296	AK Scientific, Inc. Cyclosporin A	99.888	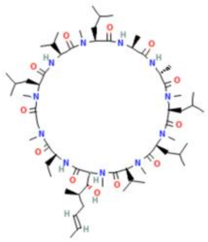
Molport-009-680-868	A2B Chem LLC Tacrolimus	99.676	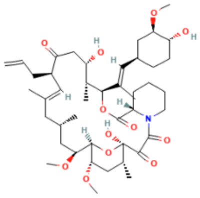
MolPort-019-901-718	ChemBridge 94417529	99.676	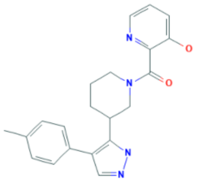
MolPort-046-108-649	ENAMINE PB2358987440	92.692	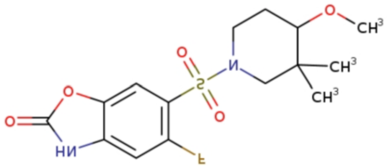
Molport-046-418-324	Combi-Blocks, Inc. Duvelisib	89.878	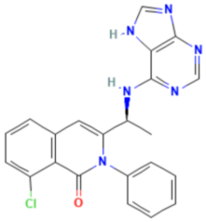
MolPort-023-141-229	ENAMINE Z1082917684	78.248	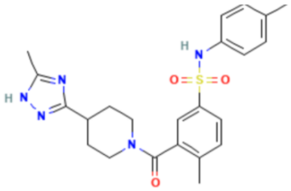

MolPort-046-143-485	ENAMINE Z646252556	73.134	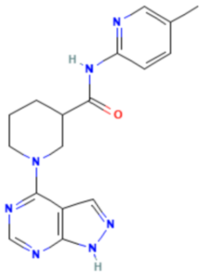
MolPort-044-691-216	ChemBridge 41652204	63.250	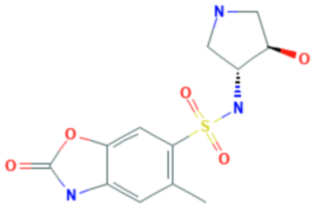
MolPort-016-593-361	ChemBridge 13786703	57.062	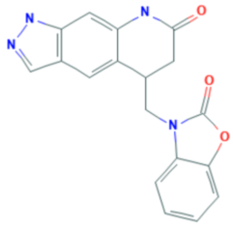
MolPort-003-138-879	ChemDiv G856-8015	55.990	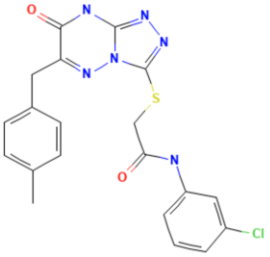
MolPort-007-841-452	ChemDiv G305-1395	54.235	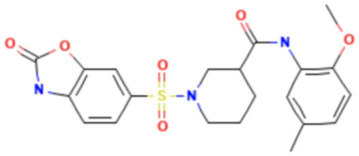
MolPort-007-688-165	ChemDiv D122-0013	52.480	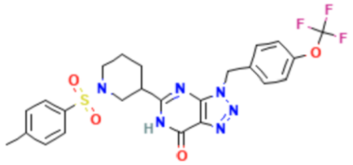

MolPort-016-626-339	ChemBridge 89912720	44.855	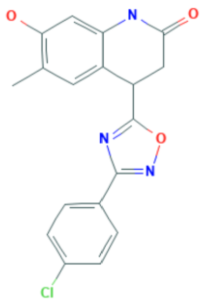
MolPort-046-727-804	ENAMINE Z3464156410	39.773	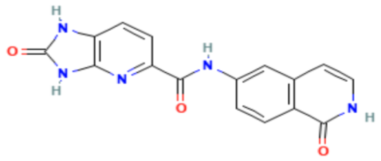
MolPort-007-688-198	ChemDiv D122-0051	38.578	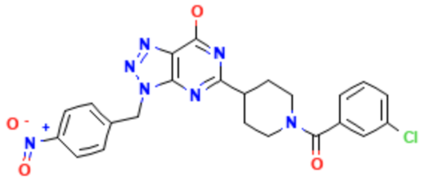
MolPort-029-998-863	Vitas-M Laboratory, Ltd. STL408477	38.027	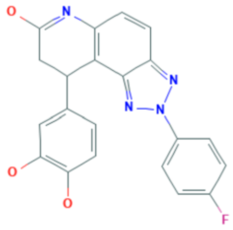
MolPort-007-696-446	ChemDiv D315-1856	32.825	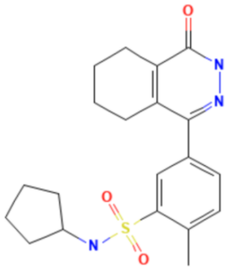
MolPort-035-823-162	Life Chemicals Inc. F6497-5780	31.706	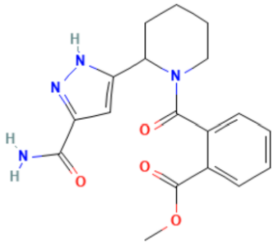

MolPort-005-708-885	ENAMINE Z229664456	5.641	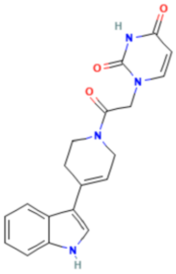
MolPort-039-119-247	ChemBridge 76995832	4.201	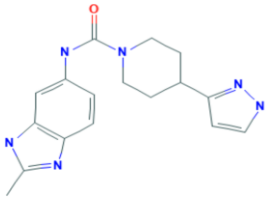
MolPort-035-717-384	L0221998	0.744	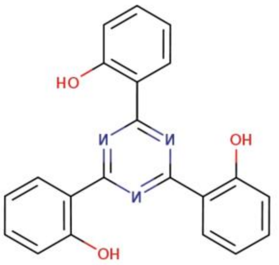
MolPort-005-727-693	ENAMINE Z253748520	<0	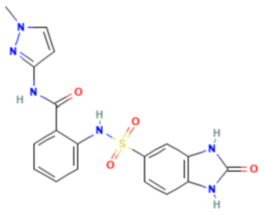
MolPort-001-584-566	Vitas-M Laboratory, Ltd. STK150021	<0	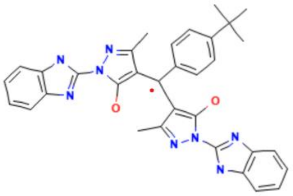
MolPort-023-175-298	ENAMINE Z1310790187	<0	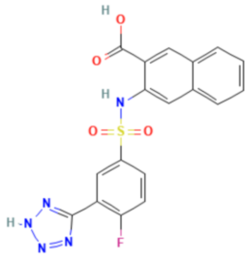
MolPort-035-851-537	ENAMINE Z1912409439	<0	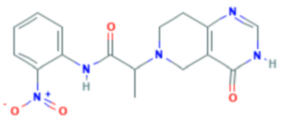

MolPort-005-807-457	ENAMINE Z124224402	<0	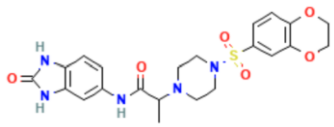
MolPort-039-032-281	Life Chemicals Inc. F6507-9155	<0	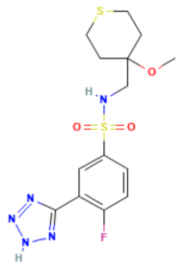
MolPort-002-737-581	Vitas-M Laboratory, Ltd. STK782471	<0	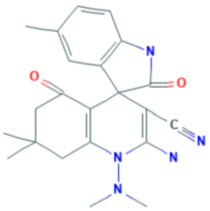
MolPort-044-598-679	ENAMINE Z2766675594	<0	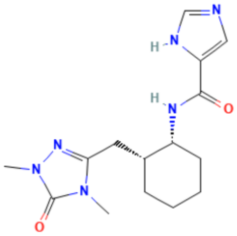
MolPort-005-916-917	Vitas-M Laboratory, Ltd. STK626358	<0	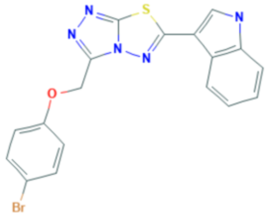
MolPort-020-210-636	ChemBridge 57034050	<0	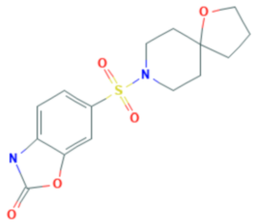

MolPort-042-601-451	ENAMINE Z2327225414	<0	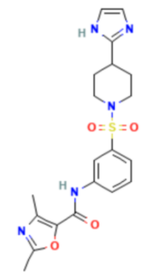
MolPort-042-504-115	ENAMINE PB2361741090	<0	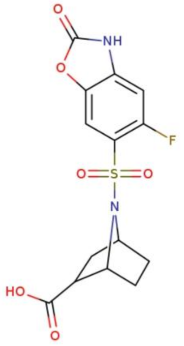

Supplemental Table 2. A comparison between Duvelisib, TG100-115, and PIK-293

Name	Molecular weight	IC50	Structure
Duvelisib<br><br>(Mishra et al., 2021)	416.87<br><br>g/mol	PI3Kγ: 50nM<br><br>PI3Kδ: 243pM	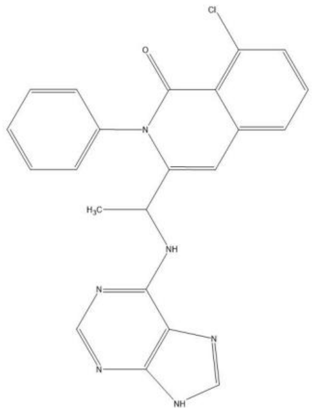
TG100-115<br><br>(Mishra et al., 2021)	346.34<br><br>g/mol	PI3Kγ: 83nM<br><br>PI3Kδ: 235nM	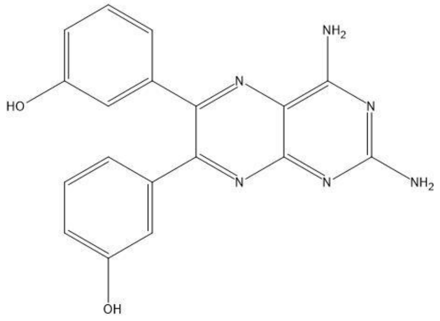
PIK-293<br><br>(Knight et al., 2006)	397.43<br><br>g/mol	PI3Kγ: 10uM<br><br>PI3Kδ: 0.24uM	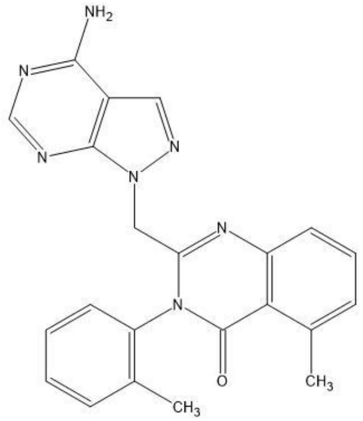

Supplemental Figure Legends

Supplemental Figure 1. Approach for the discovery of novel NFAT inhibitors and their mechanism of action. A structure-based virtual screen was conducted to identify small molecules that interact with NFATC2, attenuate its transcriptional activity, and reduce anti-adalimumab antibody formation and immunogenicity. The mechanism of action of the lead compound was investigated by evaluating its effects on calcineurin phosphatase activity, NFAT nuclear localization, and interference with NFAT-DNA interactions.

Supplemental Figure 2. Simulation of unbound NFATC2. (A) The DNA binding Rel homology region (RHR) from PDB 1OWR consists of two distinct immunoglobulin sub-domains (N-terminal: RHR-N, C-terminal: RHR-C) connected by a flexible linker. These domains are highly mobile with respect to each other as visualized by 10 snapshots from the simulation and (B) quantified in a frame-to-frame pairwise RMSD heatmap. (C) This mobility results in large root mean squared fluctuations. As the simulation progresses the protein (D) becomes more compact and (E) less solvent exposed.

Supplemental Figure 3. Simulation of NFATC2 bound to DNA. (A) The DNA binding Rel homology region (RHR) from PDB 1OWR consists of two distinct immunoglobulin sub-domains (N-terminal: RHR-N, C-terminal: RHR-C) connected by a flexible linker. These domains are stabilized by DNA and only adopt two distinct conformations during the simulation as (B) quantified in a frame-to-frame pairwise RMSD heatmap. (C) Most of the conformational change is in the RHR-C sub-domain, as evidenced by larger root mean squared fluctuations. As the simulation progresses the protein (D) becomes more compact and (E) less solvent exposed.

Supplemental Figure 4. Effect of PI3K inhibitors with and without GSK3β inhibition on Jurkat cell viability. Jurkat cells were used to assess cell viability after treatment with various PI3K-δ and PI3K-γ inhibitors (10 μM), including duvelisib (DV), TG100-115, and PIK-293, with and without the GSK3β inhibitor tideglusib (6.25 to 25 μM). The GSK3β inhibition was to determine if the effects of DV are due to GSK3β activation from PI3K inhibition. Our results show a modest impact of PI3K inhibitors on cell viability and negligible changes with tideglusib treatment up to 25 μM.

Supplemental Figure 5. A Cellular Thermal Shift Assay (CETSA) demonstrates that DV provides thermal stability to NFAT, indicating their interaction. Jurkat cell lysates were treated with 1 mM DV or vehicle (2.5% DMSO) and heated at temperatures ranging from 40-85°C. Our Western blot results show that DV substantially protects NFATC2 from heat-induced degradation and modestly increases the thermal stability of NFATC1. However, the effect of DV is specific to NFAT, as it does not provide any protective effect for β-actin.

Supplemental Figure 1

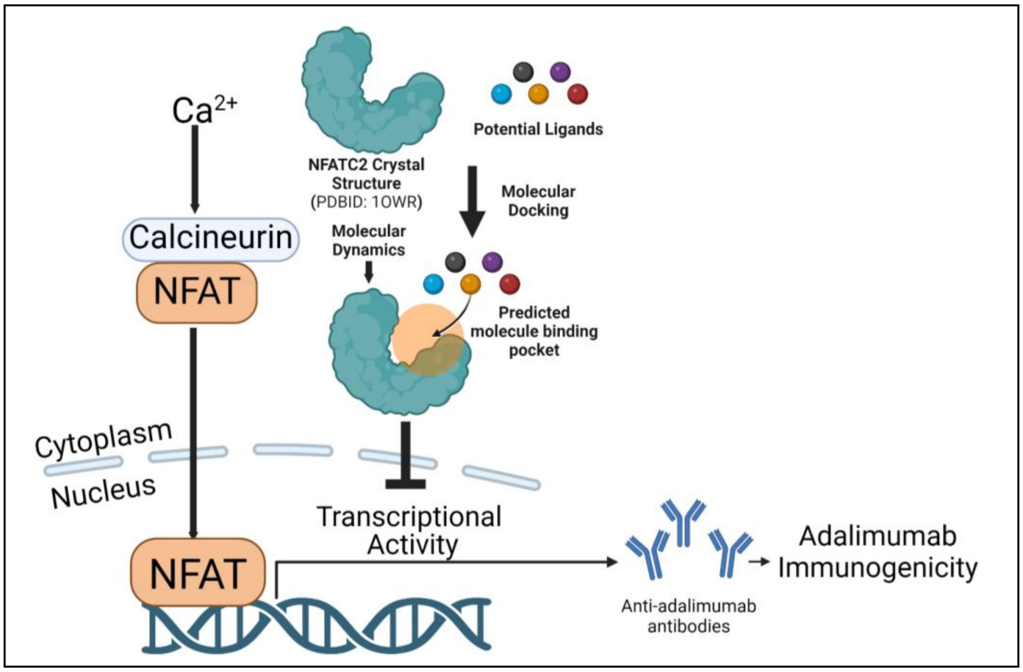

Supplemental Figure 2

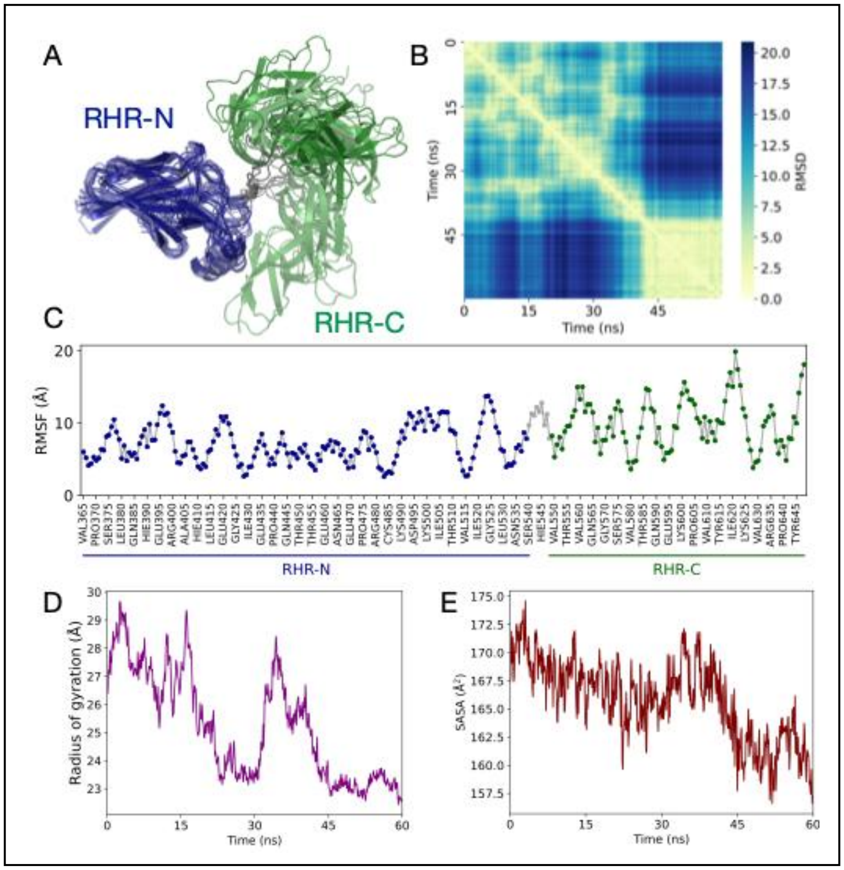

Supplemental Figure 3

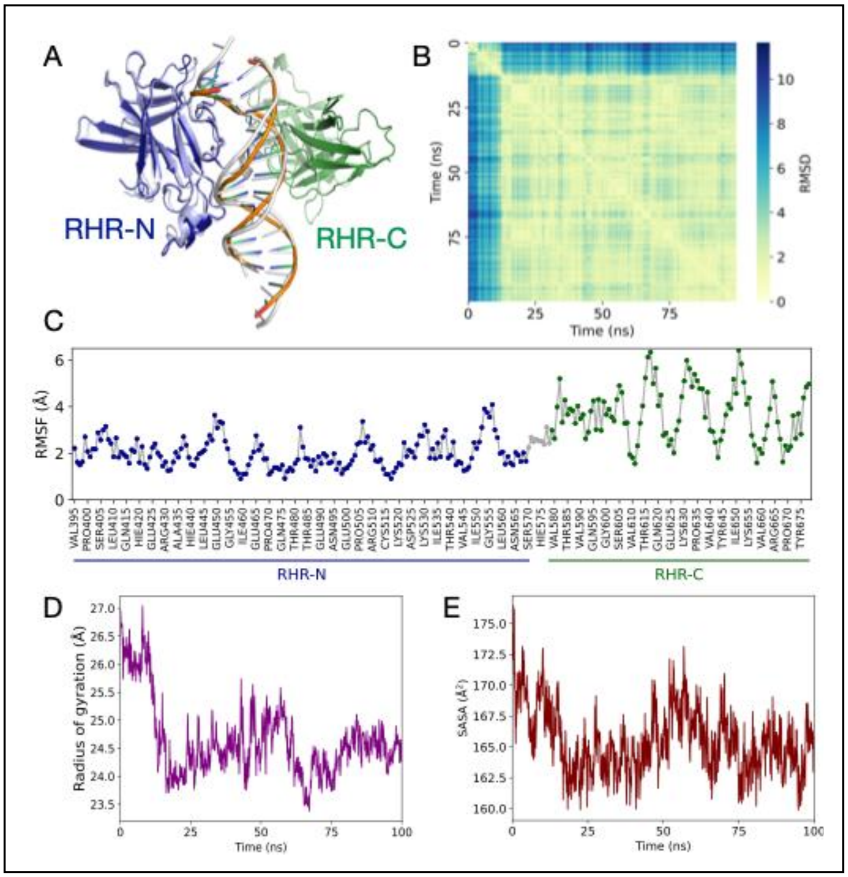

Supplemental Figure 4

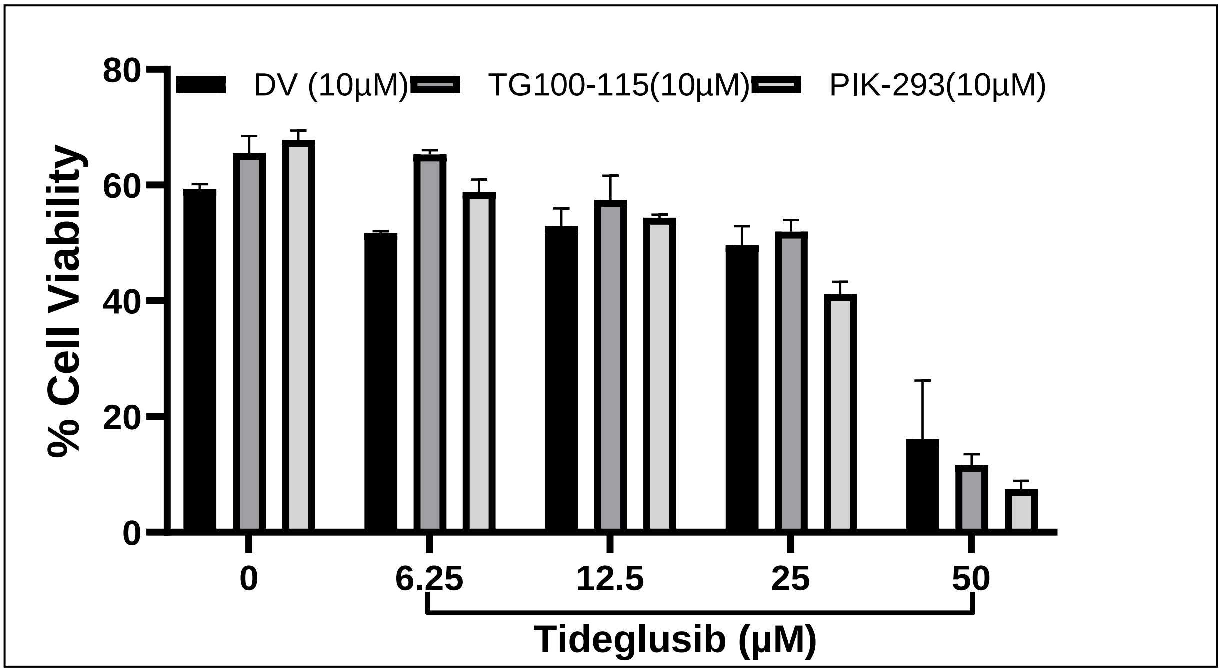

Supplemental Figure 5

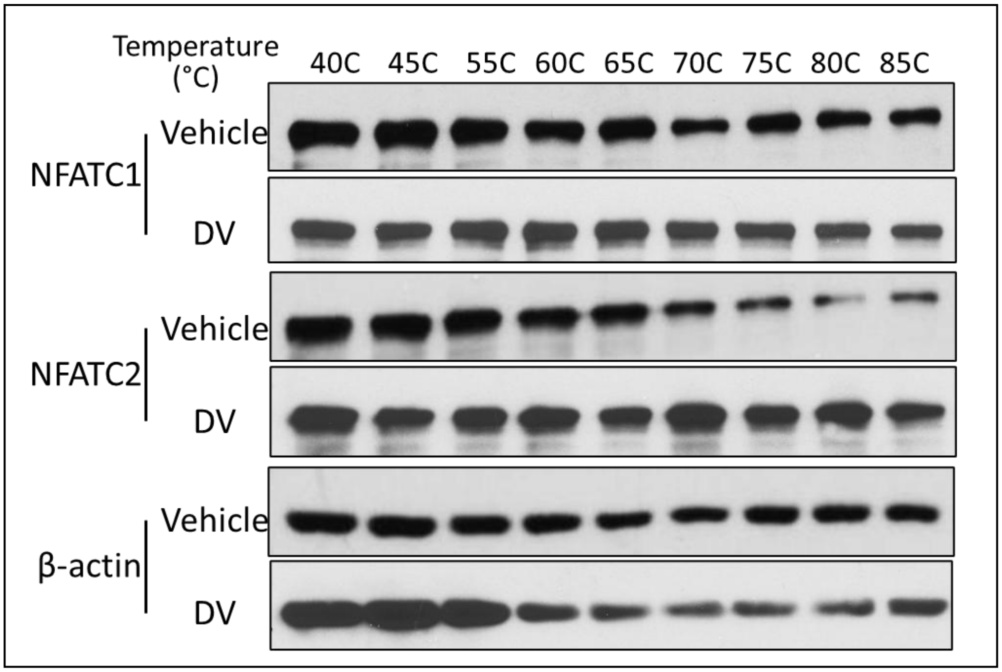

REFERENCES

Dayalan Naidu, S., Dikovskaya, D., Moore, T.W., and Dinkova-Kostova, A.T. (2022). Detection of thermal shift in cellular Keap1 by protein-protein interaction inhibitors using immunoblot- and fluorescence microplate-based assays. STAR Protoc 3, 101265.

Knight, Z.A., Gonzalez, B., Feldman, M.E., Zunder, E.R., Goldenberg, D.D., Williams, O., Loewith, R., Stokoe, D., Balla, A., Toth, B., Balla, T., Weiss, W.A., Williams, R.L., and Shokat, K.M. (2006). A pharmacological map of the PI3-K family defines a role for p110alpha in insulin signaling. Cell 125, 733-747.

Mishra, R., Patel, H., Alanazi, S., Kilroy, M.K., and Garrett, J.T. (2021). PI3K Inhibitors in Cancer: Clinical Implications and Adverse Effects. Int J Mol Sci 22.

Wang, J., Wu, J., Li, X., Liu, H., Qin, J., Bai, Z., Chi, B., and Chen, X. (2018). Identification and validation nucleolin as a target of curcumol in nasopharyngeal carcinoma cells. J Proteomics 182, 1-11.
